# ClinicalTrials.gov as a Data Source for Semi-Automated Point-Of-Care Trial Eligibility Screening

**DOI:** 10.1371/journal.pone.0111055

**Published:** 2014-10-21

**Authors:** Pascal B. Pfiffner, JiWon Oh, Timothy A. Miller, Kenneth D. Mandl

**Affiliations:** 1 Boston Children's Hospital Informatics Program, Boston, Massachusetts, United States of America; 2 Department of Pediatrics, Harvard Medical School, Boston, Massachusetts, United States of America; 3 Wellesley College, Wellesley, Massachusetts, United States of America; 4 Center for Biomedical Informatics, Harvard Medical School, Boston, Massachusetts, United States of America; CSIR-Institute of Microbial Technology, India

## Abstract

**Background:**

Implementing semi-automated processes to efficiently match patients to clinical trials at the point of care requires both detailed patient data and authoritative information about open studies.

**Objective:**

To evaluate the utility of the ClinicalTrials.gov registry as a data source for semi-automated trial eligibility screening.

**Methods:**

Eligibility criteria and metadata for 437 trials open for recruitment in four different clinical domains were identified in ClinicalTrials.gov. Trials were evaluated for up to date recruitment status and eligibility criteria were evaluated for obstacles to automated interpretation. Finally, phone or email outreach to coordinators at a subset of the trials was made to assess the accuracy of contact details and recruitment status.

**Results:**

24% (104 of 437) of trials declaring on open recruitment status list a study completion date in the past, indicating out of date records. Substantial barriers to automated eligibility interpretation in free form text are present in 81% to up to 94% of all trials. We were unable to contact coordinators at 31% (45 of 146) of the trials in the subset, either by phone or by email. Only 53% (74 of 146) would confirm that they were still recruiting patients.

**Conclusion:**

Because ClinicalTrials.gov has entries on most US and many international trials, the registry could be repurposed as a comprehensive trial matching data source. Semi-automated point of care recruitment would be facilitated by matching the registry's eligibility criteria against clinical data from electronic health records. But the current entries fall short. Ultimately, improved techniques in natural language processing will facilitate semi-automated complex matching. As immediate next steps, we recommend augmenting ClinicalTrials.gov data entry forms to capture key eligibility criteria in a simple, structured format.

## Introduction

Recruiting patients to clinical trials is an expensive, time consuming, and increasingly difficult process [1]–[6]. High market penetration of electronic medical records (EMR) presents an opportunity to integrate clinical trial eligibility evaluation into clinical workflows [7]. The physician at the point of care could be productively engaged in trial recruiting [8], for example by being alerted when a patient might be suitable for a trial. Further, a clinician seeking a trial for her patient should be readily presented with an array of appropriate trials. Because the data in the EMR contains many elements of eligibility criteria (age, gender, diagnoses, laboratory data), an application that accesses EMR data could help automate screening for clinical trials [9]. However to be integrated into daily clinical workflow the process would need to be highly efficient and streamlined. There currently is no standardized workflow or toolkit to perform such automated eligibility screening.

The new focus under the Patient Affordable Care Act on a national-scale pragmatic trial infrastructure, in which patients are randomized in trials within the delivery system, heightens the importance of efficient point-of-care eligibility screening.

ClinicalTrials.gov (CTG) [10], maintained by the United States National Library of Medicine (NLM), is the largest registry of clinical trials [11]. It has achieved a high rate of prospective trial registration for interventional trials, largely attributable to the 2005 registration requirement instituted by the International Committee of Medical Journal Editors (ICMJE) [12] and the 2007 requirement to register trials for Food and Drug Administration approval [13]. The data captured in CTG includes medical information, such as the trial's purpose, interventions and eligibility criteria, organizational information such as timeframes, sponsors and participating centers as well as basic results of completed trials.

Using CTG as a source of ground truth for trial eligibility would obviate the need to maintain separate trial databases. CTG could become an engine not only of trial registration, but also enrollment. The database could be queried at the point of care and eligibility criteria compared to EMR data and physician and patient input. In fact, several academic institutions and companies are already using CTG to provide customized trial suggestions, but those solutions are primarily designed to be used directly by the patient [14] and usually limited to a specific disease domain [15]–[19].

However, to repurpose CTG to efficiently serve as source for trial eligibility data, new processes for collecting said data may be necessary. Reliable eligibility criteria to support eligibility screening or even automated matching must be both accurate and “readable” by a computer. We sought to evaluate the utility of CTG for trial eligibility screening. We assessed:

- accuracy of trial recruitment status, that is whether a trial is still recruiting patients when its recruitment status is set to “open”. Reliable recruitment status is needed so only trials that are still open for recruitment are suggested after a first filtering step.
- semantic characteristics of patient eligibility criteria with respect to automated text interpretation through natural language processing (NLP). Structured data in CTG includes age, gender and whether healthy volunteers are being accepted and can be readily interpreted computationally. However, the remaining majority of eligibility criteria in CTG are expressed as free text and require computerized interpretation [20]. Such interpretation by means of NLP is an active area of research and several key issues are being addressed, of which we chose 3 to predict feasibility of automated criteria interpretation: defined subpopulations; laboratory values and scores [21]; and temporal constraints [21], [22]. Additionally we looked at how many criteria require specific patient action or abilities and are thus unamenable to computational evaluation.
- adequacy of contact details for the trial coordinator.

We sought to define parameters that would guide further development of automated and semi-automated clinical trial eligibility screening and matching tools and approaches.

## Methods

### Data Retrieval

The data source is CTG, listing 160,552 clinical trials from 185 countries as of February 5, 2014. We accessed CTG data using a specialized application programming interface (API) adding location data (latitude and longitude accurate to the city level) for the individual recruitment centers of each trial [23]. Scripts to facilitate data retrieval and analysis were written in the Python programming language and made openly available on our GitHub repository [24].

### Trial Set

Our goal was to create a diverse sample of trials to not limit our analysis to a certain field of study. We extracted trials open for recruitment by querying CTG for: the antineoplastic medication imatinib mesylate (“Gleevec”); “cataract”; “neuroblastoma”; and “rheumatoid arthritis”.

Exploratory searches revealed approximately 100 trials for three of the clinical areas, but over 400 for rheumatoid arthritis. Hence we randomly selected 25% of the rheumatoid arthritis trials to develop comparably sized sets.

### Recruitment Status

Several elements are in structured fields. Trial identification number, date first entered, date last updated, and study completion date were captured. The study completion date is defined as the final date on which data was (or is expected to be) collected. Trials listing a recruitment status of “Not yet recruiting” or “Recruiting” but specified a completion date in the past were marked as “conflicting”.

### Eligibility Criteria

Plain text eligibility criteria of all trials in our four sets were evaluated for the following properties:

- Do criteria adhere to the CTG recommended macro-format, in which they are formatted as a list, preceded by the words “Inclusion Criteria” and “Exclusion Criteria”, in that order?
- Are subpopulations defined? In other words are there different recruitment groups for different study arms or do certain criteria apply only to specific patients (e.g. “If patients are receiving oral corticosteroids, then…”), or are there different properties or lab ranges depending on the patient's demographics or precise diagnosis.
- How many laboratory values and medical scores do the criteria require?
- Are the criteria temporally constrained? E.g. “at least 2 weeks since therapy with drug”, “no more than 2 months since intervention” or “diagnosis of disease ≤3 years”.
- How many criteria rely on patient behavior or abilities? E.g., criteria stating “patient must be willing to comply with radiation safety procedures” or “able to walk on treadmill or cycle on a stationary bike.”

These structural elements of eligibility relate directly to the ability to use NLP techniques to parse and automatically interpret the text [25]. Where patient behavior and preferences come into play, physician or patient involvement in eligibility screening must complement automation.

### Contact Information

Contact Information from CTG was validated in a sample of representatives from 40 trials in each set. One of the authors (JO) reached out by phone or email and the representative was asked whether the trial center was still recruiting patients and, if not, when recruitment had ended.

The sets were created as follows: all trials with at least one trial location in the United States, specifically marked as “recruiting” or “not yet recruiting”, were collected per clinical area and ordered by date last updated. Each resulting list was divided into 4 segments. From each segment 10 trials were randomly selected using a Python script and the 4 segments were again combined into one list of 40 trials per clinical area. Individual trial locations of each trial were then ordered by distance to Boston, Massachusetts. The closest three locations with their contact data plus the trial's overall and backup contact data was stored in a spreadsheet.

A workflow for phone and email follow up was created that included a maximum of 3 phone calls and 1 email per trial location, starting with the geographically closest location, moving on to the other 2 locations and resorting to the overall contact information depending on contact information availability (Figure S1). The time window allowed for reply to our inquiry emails was 2 weeks.

Responses were classified into 4 categories: “open”, “closed”, “don't know” or “no answer”. The “no answer” category also applied to trials that did not supply any contact information while those who refused to answer our inquiry were categorized as “don't know”.

### Analysis

Results are described primarily with descriptive statistics. Correlation between recruitment status and the date last updated was assessed with one-sided Mann-Whitney U-tests using R 3.0.2 [26] on Mac OS X 10.9.

## Results

**Figure 1:**
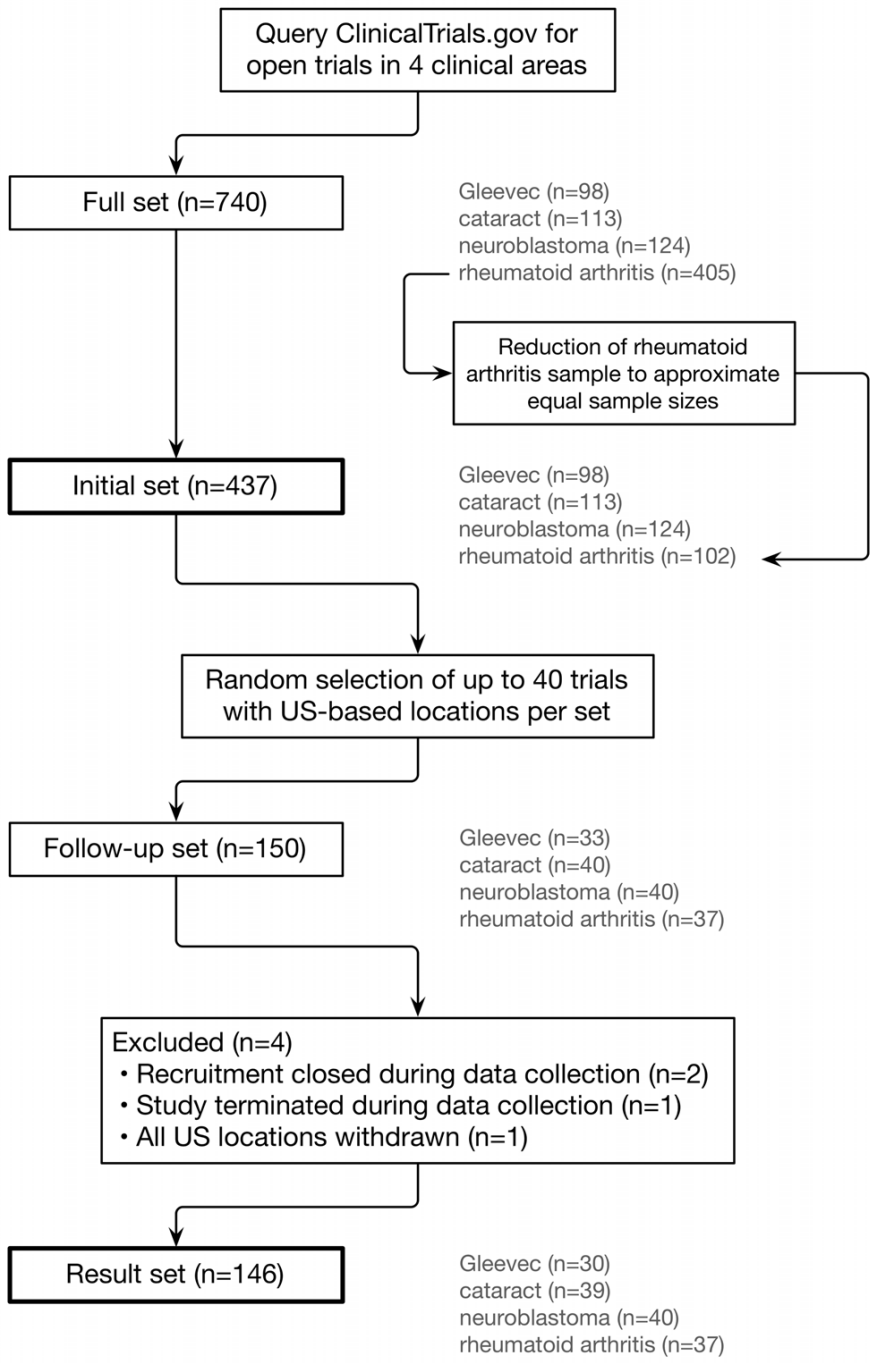
Trial selection workflow.

Figure 1 provides an overview of trial selection. Table 1 lists the number of trials for each of the clinical areas and the date data were downloaded from CTG. Our full data set of eligibility criteria evaluation consisted of 437 trials with 5,950 individual inclusion and exclusion criteria.

**Table 1:** Number of trials and eligibility criteria.

domain	date retrieved	# trials	# eligibility criteria
Gleevec	June 6, 2013	98	1641
cataract	June 12, 2013	113	1163
neuroblastoma	June 20, 2013	124	1979
rheumatoid arthritis	July 5, 2013	102	1167

Number of trials and individual eligibility criteria in our 4 data sets and the date of data retrieval from ClinicalTrials.gov.

### Recruitment Status

**Figure 2:**
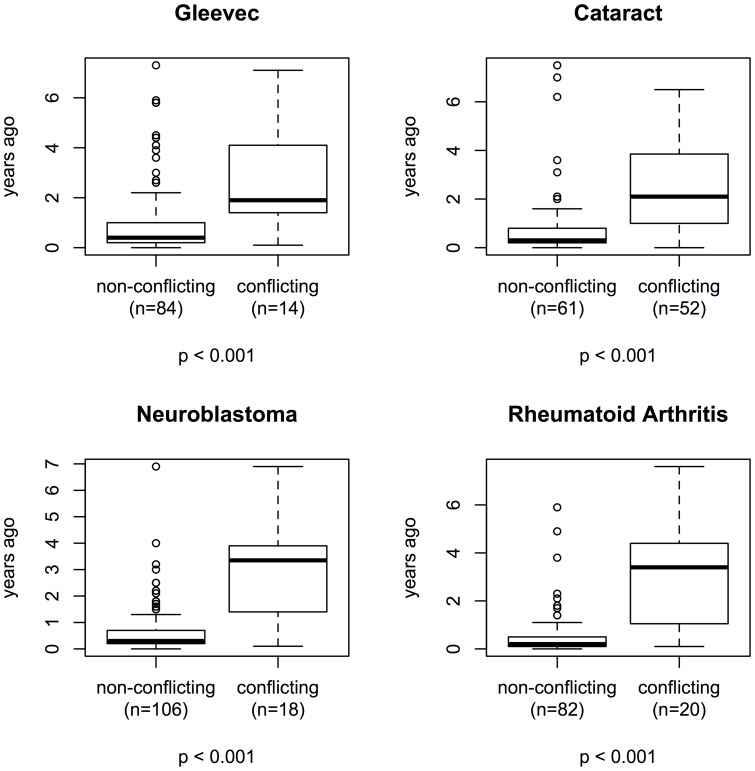
Comparison of the time since last update to whether the recruitment status is conflicting with the stated study completion date. Trials with a recruitment status conflicting with their stated completion date tended to not have been updated recently (p<0.001).

We prompted CTG to only return trials recruiting or not yet open for recruitment. A number of trials returned as “recruiting” hadn't been updated in years and some of the trials listed a completion date in the past. Thus we determined how many trials were listing an open recruitment status conflicting with a completion date in the past. Nearly half, 46% of the cataract trials, had a conflicting recruitment status, compared to 14% for the Gleevec, 15% for the neuroblastoma and 19% for the rheumatoid arthritis sets. We then compared recruitment status conflicts to the time passed since the trial was last updated on CTG. Trials with conflicting recruitment status tended to not have been updated recently (Figure 2).

### Eligibility Criteria

#### Textual Format

A majority of trials (90%) followed the CTG-suggested macro format for inclusion and exclusion criteria. There were notable differences between the sets however, the lowest being 79% for the neuroblastoma trials, then 88% for Gleevec trials to up to 98% for cataract and rheumatoid arthritis trials (Table 2).

**Table 2:** Eligibility text format.

domain	suggested format	with extended format
Gleevec	88%	97%
cataract	98%	99%
neuroblastoma	79%	97%
rheumatoid arthritis	98%	98%

Percentage of trials, per trial set, that adhere to the format suggested by CTG and the percentage of trials that either adhere to the suggested format or a second format, often found in cancer trials.

Notably, many of the noncompliant studies expressed eligibility criteria in an alternative format, listing only inclusion criteria – potentially negated – under the topics “disease characteristics”, “patient characteristics” and “prior concurrent therapy”. This alternative format could be accounted for in software and would raise the percentage of criteria suitable for automated interpretation to 97% for the Gleevec, 99% for the cataract and 97% for the neuroblastoma trials, resulting in an overall percentage of 98%.

#### Computational Barriers

Next we quantified occurrences of three categories of linguistic constructs difficult for NLP technologies to interpret correctly and whether criteria required patient actions or abilities (Table 3).

**Table 3:** Computationally challenging elgibility criteria.

domain	sub-populations	labs and scores	temporal	patient	any
Gleevec	42%	70%	78%	61%	94%
cataract	12%	48%	32%	54%	81%
neuroblastoma	35%	73%	65%	49%	90%
rheumatoid arthritis	17%	45%	64%	47%	84%

The percentage of trials, broken down per trial set, that include at least one criterion only applying to a sub-population (sub-population) of the targeted patient cohort, that contain at least one laboratory value or medical score (labs and scores), that have at least one criterion that is temporally constrained (temporal), that have at least one criterion describing patient behavior or abilities (patient) and that have at least one of these four criteria (any).

Subpopulations: When a trial specifies eligibility criteria that only apply to a subset of the study population, algorithms face additional challenges. We found that between 12% (cataract) and 42% (Gleevec) of trials specified at least one sub-population.

Laboratory Values and Scores: Between 45% (rheumatoid arthritis) and 73% (neuroblastoma) of trials contained at least one laboratory value or medical score.

Temporal Restrictions: Another difficulty for text interpretation is extracting information in the context of a temporal constraint. As Table 3 shows, three of our four sets had at least one temporal constraint in ⅔ or more of their trials. Only in the cataract set less than ⅓ of all trials had temporal constraints.

Patient Behavior and Abilities: Between 47% (rheumatoid arthritis) and 61% (Gleevec) of trials contained at least one criterion that a patient is able or willing to adhere to a given condition, necessitating patient input in a screening algorithm.

Overall, between 81% (Cataract) and 94% (Gleevec) of the trials contained at least one of these barriers to automated eligibility determination.

### Contact Information

We followed up with trial coordinators of 40 trials per set and asked about their recruitment status. Our selection method – trials that had at least one US location and were still marked as recruiting – resulted in 33 Gleevec, 40 cataract, 40 neuroblastoma and 37 rheumatoid arthritis trials.

We dropped 4 trials from our follow-up sets: three trials did complete recruitment or terminate the trial in the time between creation of our data sets and downloading contact data, which makes contact data unavailable via CTG. One trial withdrew all US locations while still maintaining non-US locations. Because we only regarded the overall recruitment status when creating our data set, this trial was erroneously included.

**Figure 3:**
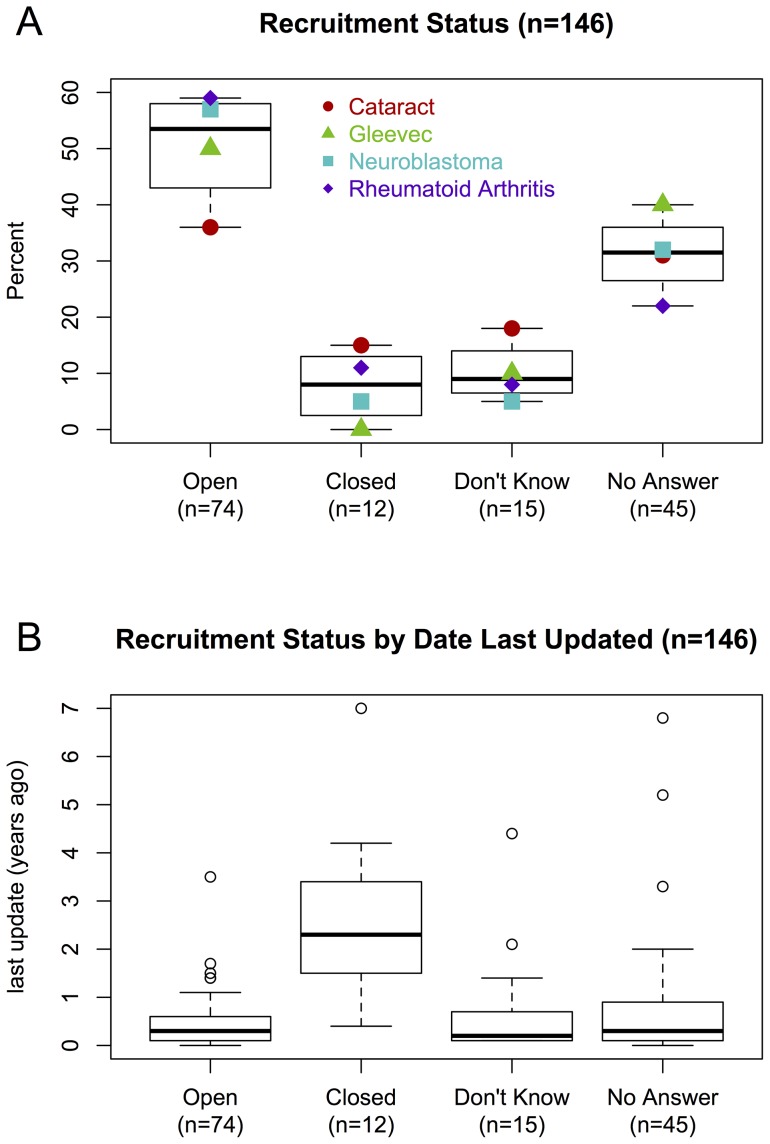
Effective recruitment status. A) Recruitment status (x-axis), verified by phone or email, of the trials in our 4 trial sets. B) The same recruitment status compared against how long ago the trials were last updated on ClinicalTrials.gov. Trials closed for recruitment tended not to have been updated recently when compared to trials open for recruitment (p<0.001).

During follow-up we were unable to contact 31% (45 of 146) of all trials (Figure 3A). These follow-ups encompassed three phone calls to the closest three recruitment locations and the contact supplied as “overall contact” to CTG as well as at least one email, depending on data availability.

Three trial centers refused to give out recruitment status information and were categorized as “don't know”. We were able to confirm that 53% (74 of 146) of trials in our sample, listed as “recruiting” on CTG, were still recruiting patients. Figure 3A breaks down the recruitment status per clinical area.

While trials open for recruitment tended to have been updated more recently when compared to trials closed for recruitment (p<0.001), there was no difference in the time since last update among the trials still open for recruitment, those that didn't know their recruitment status and trials at centers that couldn't be contacted (Figure 3B).

## Discussion

With a goal of assessing feasibility of robust, semi-automated screening of patients' eligibility for clinical trials we evaluated sample aspects of data quality in CTG registrations. We identified several key issues.

Nearly a quarter of trial records were notably out of date, in that they listed as “open for recruitment” but stating a study completion date in the past. Out-of-date registrations are an issue previously reported in a study comparing registry data to study protocols [27].

Further, most trials adhere to one of two textual formats for eligibility criteria, represented as unstructured text. Computational processing of such data thus requires a preliminary data extraction step, which poses an obstacle to automation. A particular difficulty is extraction of laboratory values expressed as free text – which occurs in about two thirds of the trials. Matching laboratory values from CTG directly to EMR data is an important opportunity.

Another complexity is understanding temporal information in the eligibility criteria. For example, “Patients must be taking MTX [Methotrexate] for at least 3 months before randomization and have to be on a stable dose at least 4 weeks before randomization.” Extracting temporal information is an active area of research in clinical NLP [28], [29], but temporal relation extraction is a difficult problem and has not yet been satisfactorily solved [30]. Thus temporal relations are likely to be lost to NLP pipelines, affecting 32% of the trials in the cataract set, similar to previously reported results of 40% [21] and 38% [22], up to 78% in the Gleevec set.

A third challenge is that many of the criteria require logical inference – both semantic understanding of the text in context and real world knowledge. For example, “Have limited disease that would not normally be treated with CYC [Cyclophosphamide]” is easily understood by a clinician but context (“limited disease”) and real world knowledge (“normally”) is a sufficiently hard problem for automated interpretation. Similarly, understanding that a textual list of criteria, indented one level deeper than the previous list item, only applies to patients with a specific diagnosis (a sub-population) requires interpretation of textual as well as structural context. Sub-populations may be present in more than a third of the trials and invalidate automated matching for a whole trial, not only a single criterion, because requirements only intended for specific patients will be applied to all patients.

More than half of trials require, for eligibility, specific patient actions and abilities. Hence there will be residual information to obtain directly from patients and clinicians beyond EMR and CTG data even in a robust semi-automated matching process.

Finally, trial status and contact are low quality fields in CTG. Only half of our trials could be contacted and would confirm that they were still recruiting patients. No contact could be established with almost one third of the trials. Trials that hadn't been updated recently were associated with a higher probability of being closed for recruitment, however the date last updated was not a predictor of whether trial recruitment centers could successfully be contacted or not.

## Conclusions and Recommendations

We suggest that a tightly integrated informatics toolkit could facilitate identification of clinical trials for which a patient might be eligible. Point-of-care apps [31], utilizing trial data sources supplemented with data from EMRs and direct clinician or patient input, can then be developed for different stakeholders such as hospitals, primary care physicians and patients. Using CTG as data source for trial eligibility evaluation is a promising approach, not least because the requirements for prospective clinical trial registration continuously increase the quantity of available trial data. However, registry data are partially out of date or inaccurate (recruitment status, contact data) and largely unstructured and therefore not readily amenable to automated data matching (eligibility criteria). Using NLP for eligibility criteria extraction from CTG holds promise but considerable obstacles must be overcome. Arriving at a formal representation of eligibility criteria that are interpretable by computer systems is an active area of research [20], [21], [25], [32], [33] and (semi-)automated systems to help translate free-text criteria into computable representations are being developed [22], [34], [35].

There are, broadly speaking, two approaches for automated trial matching based on CTG data:

1. Working with the data already contained in CTG, enhanced with NLP technologies and/or manual curation to arrive at computable eligibility criteria representations.
2. Extend and standardize the eligibility data contained in CTG to additionally contain structured eligibility data.

The first approach bumps up against the cutting edge of NLP technology and may create services that are not universally available. The second approach relies on an accepted standard for formal eligibility criteria representation and shifts additional burden to CTG, which needs to provide additional data fields, and data providers who need to enter and maintain additional registry data. We contend that data providers may be motivated to do so based on the value proposition of accelerated accrual in return.

However, there might be a middle ground to enable initial eligibility screening at a finer level than is currently possible. Allowing data providers to specify a handful of key eligibility criteria in structured form, in addition to age and gender, might provide data for very powerful initial trial filtering. Such key criteria would vary between clinical specialties and could include “taking Methotrexate”, “no previous eye surgery” or even simple laboratory value ranges. They ideally would not require an extensive format specification but could rely on existing coding systems such as SNOMED-CT and RxNorm, coupled with boolean logic or numeric ranges.

Though an extensive modeling effort to represent all clinical trial eligibility criteria in structured form could lead to a prolonged consensus process, targeting the “low hanging fruit” first – by adapting existing data standards – would be a straight forward and cost effective approach to improving automated trial matching. Leaders of clinical trials would be presented with an attractive value proposition – keeping their CTG records accurate and up to date might promote improved and timelier accrual.

Importantly, substantial investment by the National Institutes of Health and the Patient Centered Outcomes Research Institute in infrastructure to implement pragmatic trials at the point of care may dramatically increase the utility of a high quality, computable, ClinicalTrials.gov-based trial data source.

## Supporting Information

Figure S1 **Phone and email follow up.**
(PDF)
