# Supplementary figures and images for: ClinicalTrials.gov as a Data Source for Semi-Automated Point-Of-Care Trial Eligibility Screening
Source: PLoS One. 2014 Oct 21;9(10):e111055. doi: 10.1371/journal.pone.0111055 (PMC4205089; doi:10.1371/journal.pone.0111055)

## Trial Status Verification

Skip locations that are:

- no longer recruiting
- not in the US

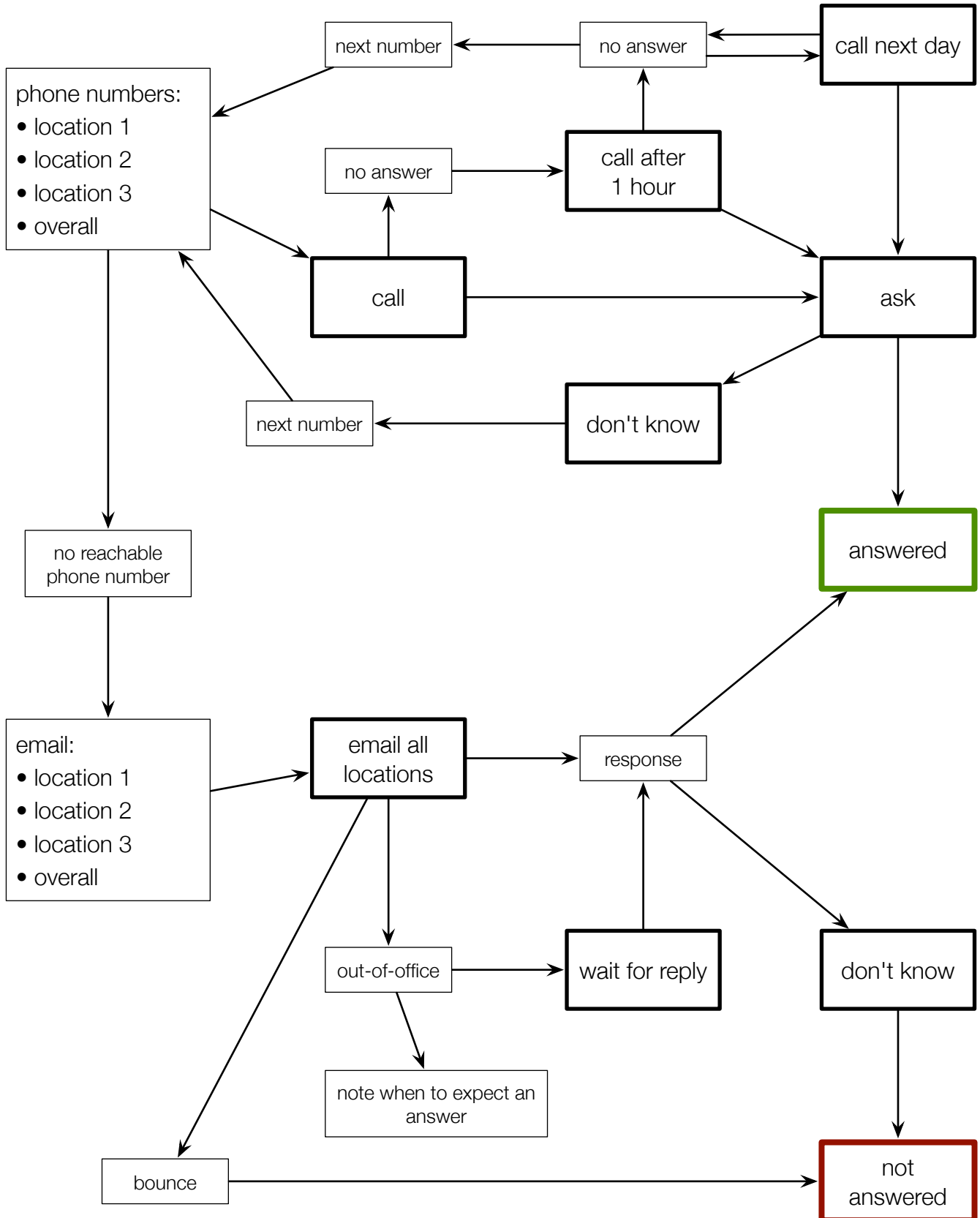

Supplement: Figure S1 — Phone and email follow up. (PDF) [file pone.0111055.s001.pdf]
